# Interfacial properties of [Pt/Co/Pt] trilayers probed through magnetometry

**DOI:** 10.1038/s41598-021-90239-x

**Published:** 2021-05-24

**Authors:** Young Chan Won, Sang Ho Lim

**Affiliations:** 1 Department of Nano Semiconductor Engineering, Korea University, Seoul, 02841 South Korea; 2 Department of Materials Science and Engineering, Korea University, Seoul, 02841 South Korea

**Keywords:** Magnetic properties and materials, Characterization and analytical techniques, Magnetic properties and materials

## Abstract

The magnetic and interface properties of [Pt/Co/Pt] were investigated. First, the magnetic properties were determined from the magnetic dead layer plots, in which the Co layer was considered as two distinct parts representing different magnetic properties. The two parts with low and high *t*_Co_ ranges are close to and away from the top interface (Co/Pt), respectively. The part close to the top interface shows a smaller magnetization (*M*) value and nonlinear behavior. However, the other part shows a higher *M* value closer to the bulk value and a linear behavior. The nonlinear behavior of the *M* values of the low *t*_Co_ range was converted to an impurity level using simple assumptions. The results showed the effect of the top Pt layer on the magnetic properties of the Co layer. The results clearly demonstrate that magnetometry could be utilized as a means to understand the interface quality of magnetic multilayer systems.

## Introduction

Recently, magnetic structures with perpendicular magnetic anisotropy (PMA) have been extensively studied, because they are essential for the development of high-density magnetic random access memory^1,2^. One of the most important materials showing a strong PMA is the [NM/Co]_n_ multilayer (where NM and n denote the nonmagnetic material and number of iterations, respectively)^3–5^. In these structures, PMA is achieved through the interface effects. However, its strength is sensitive to the flatness and atomic intermixing of the interfaces^6,7^. The interface flatness can be improved by designing proper seed/buffer layers before depositing the multilayers. However, the atomic intermixing is extremely difficult to prevent if the stacks are fabricated through sputtering, in which the sputtered particles arriving at the substrate are rather energetic^8^. The [Pt/Co] multilayer system, which is one of the most popular multilayer systems exhibiting a strong PMA, can be composed of two different types of interfaces: Pt (bottom)/Co and Co (bottom)/Pt. Considering that Pt is significantly heavier and more strongly bonded than Co, more intermixing will occur at the Co/Pt interface than at the Pt/Co interface^9–11^. Therefore, to develop materials with a strong PMA, it is important to minimize the intermixing at the Co/Pt interfaces. Some efforts have been made in this direction, which include inserting a spacer layer such as Cu between Co and Pt^12^ and reducing the Pt layer thickness down to 0.2–0.25 nm^3–5^. The latter results in [Pt/Co] multilayers with an inverted structure in which Pt is thinner than Co; this showed a strong PMA and high post-annealing stability^4,5^. Very recently, a model system with a trilayer structure of [Pt/Co/Pt] was investigated for a more comprehensive understanding of the roles played by the Pt/Co and Co/Pt interfaces in influencing the PMA strength^11^. The PMA strength due to the top Co/Pt interface was found to be significantly weaker than that of the bottom Pt/Co interface. A similar difference in the interface quality was expected between the two interfaces but no evidence was found. In this study, conventional magnetometry was used to analyze the interface quality of the bottom Pt/Co and top Co/Pt interfaces in the [Pt/Co/Pt] trilayers; however, a more emphasis was placed on the latter, because its interface quality and PMA strength are greatly affected by annealing and the top Pt thickness^4,5,11^.

## Results

### Microstructural characterization by HRTEM

**Figure 1:**
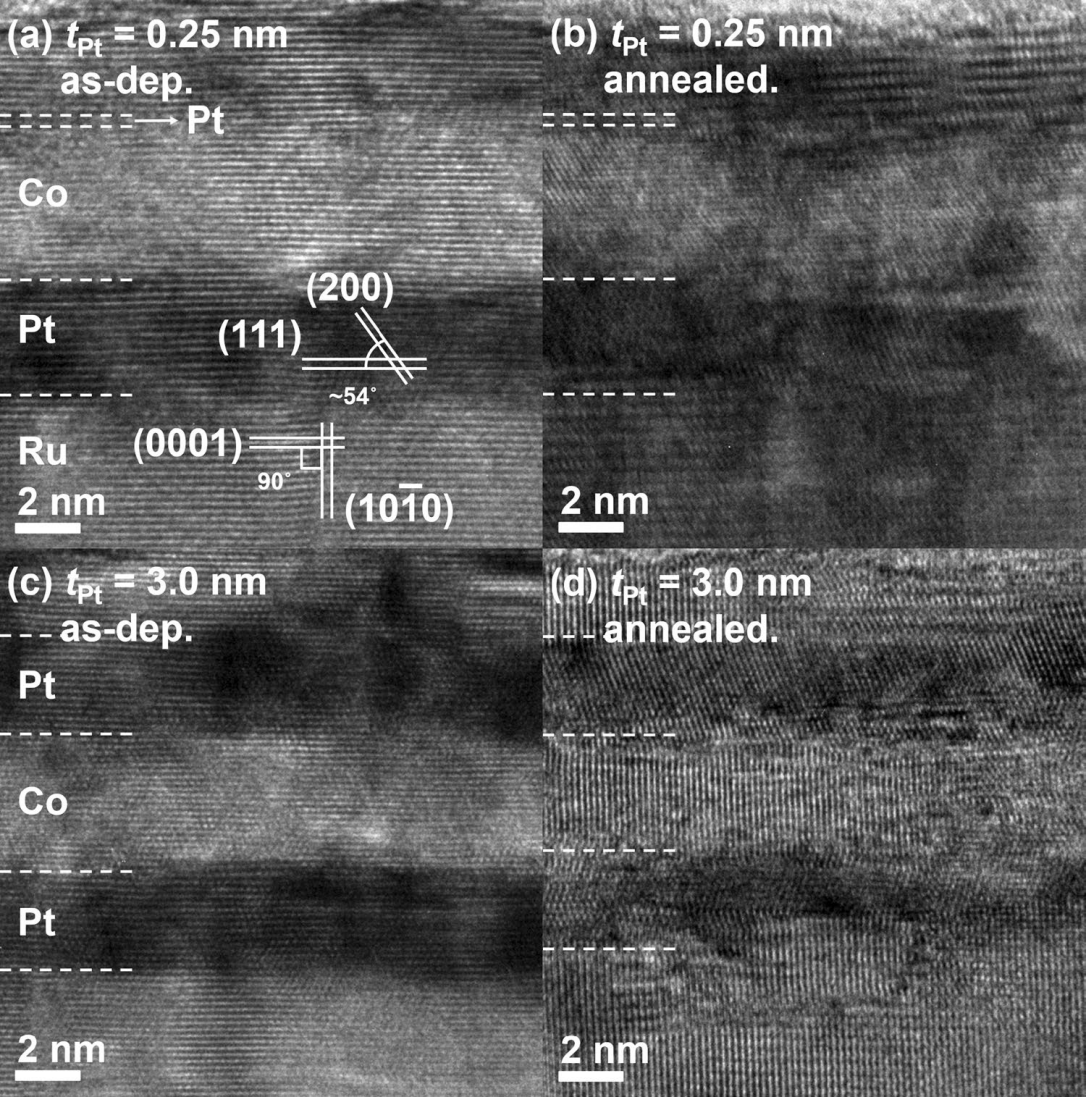
Cross-sectional HRTEM images of the [Pt/Co/Pt] structures with two *t*_Pt_ values in both as-deposited and annealed samples. Results of the samples with (**a**,**b**) *t*_Pt_ = 0.25 nm and (**c**,**d**) *t*_Pt_ = 3.0 nm. Results for the (**a**,**c**) as-deposited samples and (**b**,**d**) annealed samples.

To examine the microstructure of the interfaces, two typical samples were chosen for HRTEM experiments: (1) *t*_Co_ = 5.0 nm and *t*_Pt_ = 0.25 nm, and (2) *t*_Co_ = 5.0 nm and *t*_Pt_ = 3.0 nm. Figure 1a–d show cross-sectional HRTEM images of these samples. The upper images (Fig. 1a,b) display the first sample (*t*_Co_ = 5.0 nm and *t*_Pt_ = 0.25 nm), whereas the lower images (Fig. 1c,d) display the second samples (*t*_Co_ = 5.0 nm and *t*_Pt_ = 3 nm). Figure 1a,c display the as-deposited samples, and Fig. 1b,d display the annealed samples. The HRTEM images show that the stacks consist of a well-developed layered structure with atomically sharp interfaces. A detailed analysis of the images indicated that the Ru seed-layer has a hexagonal close-packed (hcp) structure and the lower Pt layer, located on top of Ru, has a face-centered cubic (fcc) structure. However, the crystalline structure of the Co layer could not be identified from the present HRTEM results because the d-spacing of hcp (0001) was nearly identical to that of fcc (111). It is rather difficult to identify a thin Pt layer (*t*_Pt_ = 0.25 nm) from the HRTEM images in Fig. 1a,b. However, our numerous results carried out on [Pt/Co] multilayers and [Pt/Co/Pt] trilayers using very thin Pt layers down to 0.2 nm^4,5,11^ clearly demonstrate that the Pt layer forms a continuous coverage and its thickness is accurate. The main aim of the HRTEM experiments was to observe the difference in the flatness and intermixing of the Pt/Co and Co/Pt interfaces as a function of *t*_Pt_ and their variation upon annealing. It was observed that the PMA strength of the top Co/Pt interface decreases upon annealing^11,13^, indicating that an intermixing occurs at the interface. However, no conclusive results could be drawn from the HRTEM images, except that the two interfaces became more blurred upon annealing. Therefore, the magnetic properties of the samples could not be adequately studied from the interface microstructural characterization.

### MDL plots

**Figure 2:**
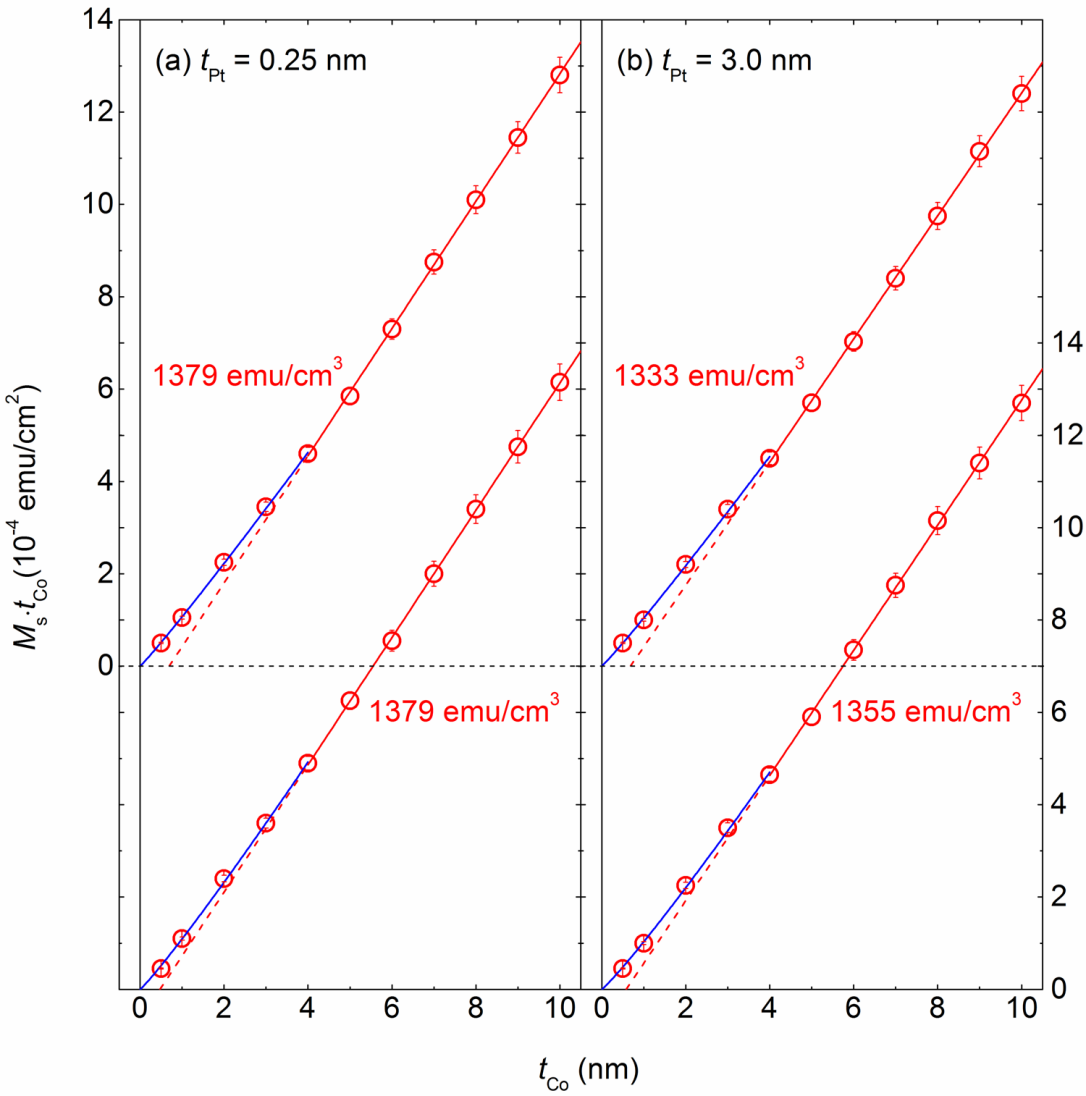
Results for *M*_s_·*t*_Co_ as a function of *t*_Co_ at (**a**) *t*_Pt_ = 0.25 nm and (**b**) *t*_Pt_ = 3.0 nm in as-deposited and annealed samples. Blue solid lines show the linear fits for the results of *t*_Co_ ≤ 4 nm, whereas red solid lines show the linear fits for the results of *t*_Co_ ≥ 4 nm; all the broken lines represent the extrapolated lines for the blue and red solid lines. The *M*_s_ values extracted from the fits are also given in the figures.

Consequently, a systematic magnetometry study was performed, which mainly involved plotting the magnetic moment as a function of *t*_Co_. These results are shown in Fig. 2a,b over a wide *t*_Co_ range for the samples with *t*_Pt_ = 0.25 and 3.0 nm, respectively. The upper set of results in each figure represents for the as-deposited samples, whereas the lower set represents the annealed samples. In these figures, not the magnetic moment itself but its normalized value according to the sample area (emu/cm^2^) is plotted as a function of *t*_Co_ so that the slope corresponds to the magnetization value. An obvious analytical equation describing the results in the high *t*_Co_ range is $y = ax + b$. Coefficient *a* is identical to the saturation magnetization value (*M*_s_ in emu/cm^3^), and the *x* value at which *y* = 0 (viz., − *b*/*a*) indicates the magnetic dead layer (MDL) thickness. Notably, the MDL thickness obtained in this way is of little physical significance because the magnetic moment is not zero at the MDL thickness. This linear behavior indicates that the *M*_s_ value can be determined in this *t*_Co_ range. For the samples with *t*_Pt_ = 0.25 nm, *M*_s_ = 1379 emu/cm^3^ for both samples in the as-deposited state and after annealing. However, for the samples with *t*_Pt_ = 3.0 nm, this *M*_s_ value is reduced substantially to 1333 emu/cm^3^ in the as-deposited state, even though it increased slightly to 1355 emu/cm^3^ after annealing. All these values are lower than those reported for bulk Co: 1422 emu/cm^3^ for hcp Co and 1450 emu/cm^3^ for fcc Co^14^. This reduction in *M*_s_ is because the thin Co layers accommodate a substantial proportion of atoms at the interfaces. Another possibility is the interpenetration of Pt atoms into the Co layer during sputtering. The lower *M*_s_ values for the samples with *t*_Pt_ = 3 nm could be attributed to the greater interpenetration for a sample with a thicker Pt layer. However, for the samples with *t*_Pt_ = 3 nm, the slight increase in *M*_s_ after annealing is unexpected because Pt atoms are likely to diffuse into the Co layer during annealing, causing a further decrease in the *M*_s_ value. This unexpected increase in *M*_s_ may indicate an opposite behavior of de-mixing during the annealing, which is likely to occur if the Co layer has an hcp structure unlike the fcc structure of the interpenetrated Pt atoms. A similar de-mixing behavior was reported for the systems of a Co–Pt alloy and [Pt/Co] multilayers, and it is contradictory to the behavior observed for the samples with *t*_Pt_ = 0.25 nm, where no change in *M*_s_ occurs upon annealing. This indicates that the interpenetration of Pt atoms into the Co layer in this case is minimal, at least in the thickness range of *t*_Co_ ≥ 4 nm.

A deviation from the linear behavior is visible in the low *t*_Co_ range. As the *t*_Co_ value decreases, all the plots show an upward deviation, which contrasts with the conventional MDL plot showing a linear behavior. Considering that the slope of the plot is identical to the *M*_s_ value, the upward deviation indicates that the *M*_s_ value in this low *t*_Co_ range decreases with decreasing *t*_Co_. This could be due to the interpenetration of nonmagnetic Pt atoms into the Co layer. A similar upward deviation was not observed in the MDL plots for [Pt/Co/Cu] trilayers^15^, indicating that the saturation magnetization of Co is constant in the Co thickness range considered in the MDL plots. As the relative portion of the interpenetrated region over the entire Co layer will increase with decreasing *t*_Co_, the observed results show a lower *M*_s_ value at a lower *t*_Co_. For quantitative analysis, significant results could be obtained using an analytical equation. Although the choice for the analytical equation in the low *t*_Co_ range is not so obvious, the following equation accurately describes the results: $y = a(x - b)^{c}$, where parameters *a*, *b*, and *c* are summarized in Table 1. In this equation, exponent *c* denotes the deviation from the linear behavior. When $c = 1$, this equation converges into its linear form for the high-*t*_Co_ region. The extracted *c* values were very close to 1, indicating that the deviation from the linear behavior is not large. For the as-deposited samples, *c* = 1.057 (*t*_Pt_ = 0.25 nm) and 1.061 (*t*_Pt_ = 3.0 nm), and these slightly increased to 1.085 (*t*_Pt_ = 0.25 nm) and 1.094 (*t*_Pt_ = 3.0 nm) for the annealed samples, indicating an increased deviation from the linear behavior after annealing. Furthermore, the *c* values for the stacks with *t*_Pt_ = 0.25 nm are smaller than those for the stacks with *t*_Pt_ = 3.0 nm for both as-deposited and annealed samples, indicating that the Pt penetration affects the magnetic properties.

**Table 1:** Summary for the parameters of the equation; $y = a(x - b)^{c}$.

	*t*_Pt_ = 0.25 nm		*t*_Pt_ = 3.0 nm	
	As-deposited	Annealed	As-deposited	Annealed
*a*	1069.6	1094.5	1042.7	1032.6
*b*	− 7.8 × 10^−11^	− 1.5 × 10^−10^	− 2.3 × 10^−9^	− 2.8 × 10^−10^
*c*	1.057	1.085	1.061	1.094

### Magnetic properties in Co layer derived from MDL plots

**Figure 3:**
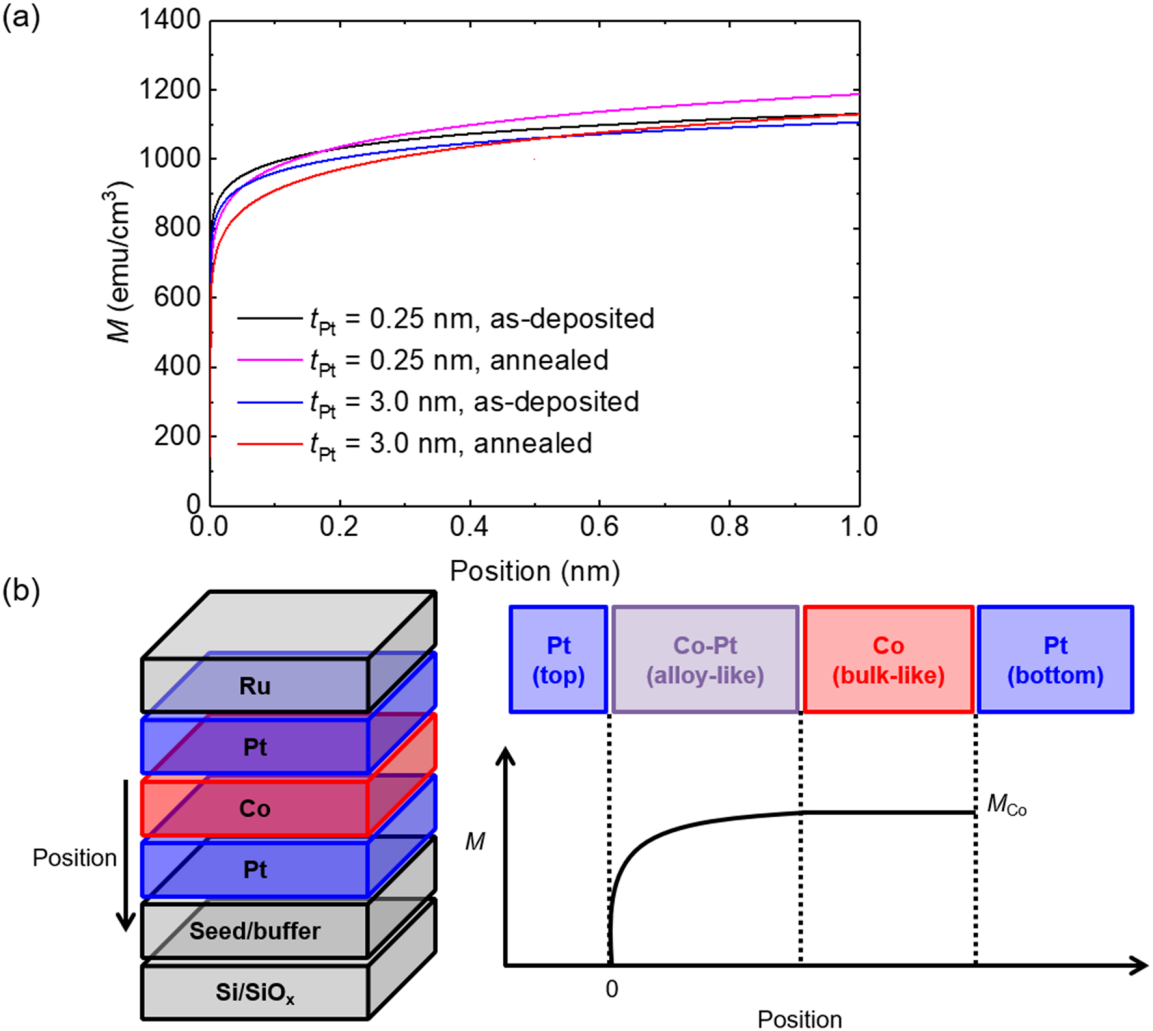
(**a**) Results for the *M* values as a function of the position in Co region at Pt thicknesses of 0.2 and 3.0 nm in the as-deposited and annealed samples. (**b**) Schematic illustration of the [Pt/Co/Pt] trilayers system (left) and the *M* values as a function of the position in the Co region (right).

Based on the analytical equations, magnetization (which is identical to the derivative of the equation, according to *t*_Co_ (*x*)) can be obtained. Figure 3a shows the results for *M* (magnetization) as a function of the position in the Co layer. The schematics of the stack structure (left) and a typical variation of *M* with the position in the Co layer (right) are shown in Fig. 3b. For the bottom Pt/Co interface, where the intermixing level during sputtering is negligible, leading to a well-defined interface, the *M* value is close to the bulk value^11^. Notably, for all the cases, *M* ≠ 0 at *t*_Co_ = 0, indicating the existence of an interface magnetization. A probable reason for the interface magnetization is that nonmagnetic Pt atoms can have a magnetic moment when they are in contact with magnetic Co atoms; this is known as the proximity effect^16,17^. Mukhopadhyay et al.^18^ reported that the induced magnetic moment does not differ significantly between the Pt/Co and Co/Pt interfaces; however, Kim et al*.*^19^ reported that the proximity effect from the top interface is stronger than that from the bottom one. In this paper, no evidence is observed on the relative strength of the proximity effect between the bottom and top interfaces. Therefore, only the amount of proximity effect is considered. The interface magnetization values were not small, i.e., the values were 301.7 emu/cm^3^ (for *t*_Pt_ = 0.25 nm) and 328.8 emu/cm^3^ (for *t*_Pt_ = 3.0 nm) for the as-deposited samples and 174.5 emu/cm^3^ (for *t*_Pt_ = 0.25 nm) and 143.5 emu/cm^3^ (for *t*_Pt_ = 3.0 nm) for the annealed samples.

These interface magnetization values due to the proximity effect can be converted into the magnetic moment possessed by one Pt atom magnetized using the following simple relation:

$$ \begin{gathered} M_{{\text{s}}} \left( {\frac{{{\text{emu}}}}{{{\text{cc}}}}} \right) \times {\text{atomic weight}}\left( {\frac{{\text{g}}}{{{\text{atom}}}}} \right) \div {\text{density}}\left( {\frac{{\text{g}}}{{{\text{cc}}}}} \right) \hfill \\ \quad = {\text{atomic magnetic moment}}\left( {\frac{{{\text{emu}}}}{{{\text{atom}}}}} \right) \hfill \\ \end{gathered} $$

The magnetic moments obtained are in the range of 0.25 to 0.54 *μ*_B_, which agree with the reported values^16,17^.

### Estimation of impurity profiles in Co layer

Based on the magnetometry results and their analysis, a schematic showing the concentration of interpenetrated impurity atoms in the Co layer can be drawn as a function of its location if the following two simplifying assumptions are made. First, intermixing during sputtering occurs only at the Co/Pt interface. Second, for the samples with *t*_Pt_ = 0.25 nm, in addition to the Pt atoms, the Ru atoms (the capping layer), deposited on the top Pt layer, can penetrate the Co layer; this is likely as the Pt thickness is low. In this case, the penetration of Ru atoms is assumed to occur during the deposition of a 2.75 nm Ru capping layer so that the total thickness (3 nm) affecting the interpenetration can be identical to that of the sample with *t*_Pt_ = 3 nm. While converting the results of *M* into the impurity concentration, it is necessary to have information on the variation of *M*_s_ with respect to the concentration of the impurities (Pt and Ru); this has been detailed in^20–22^ for Pt and in^23^ for Ru. A simple linear assumption in the required composition range is considered reasonable for the following two reasons. First, Co can have an fcc structure for a very thin Co layer. Therefore, Co atoms are likely to be miscible with Pt with an fcc structure^24–26^. Second, the Co–Ru binary phase diagram indicates that the atoms are miscible in the range up to 6 at.% of Ru. An equation used for the conversion is as follows.

$$ M_{{{\text{s}},{\text{Co}}}} - M_{{{\text{s}},{\text{sample}}}} = A_{{{\text{impurity}}}} \times C_{{{\text{imputiry}}}} $$

**Figure 4:**
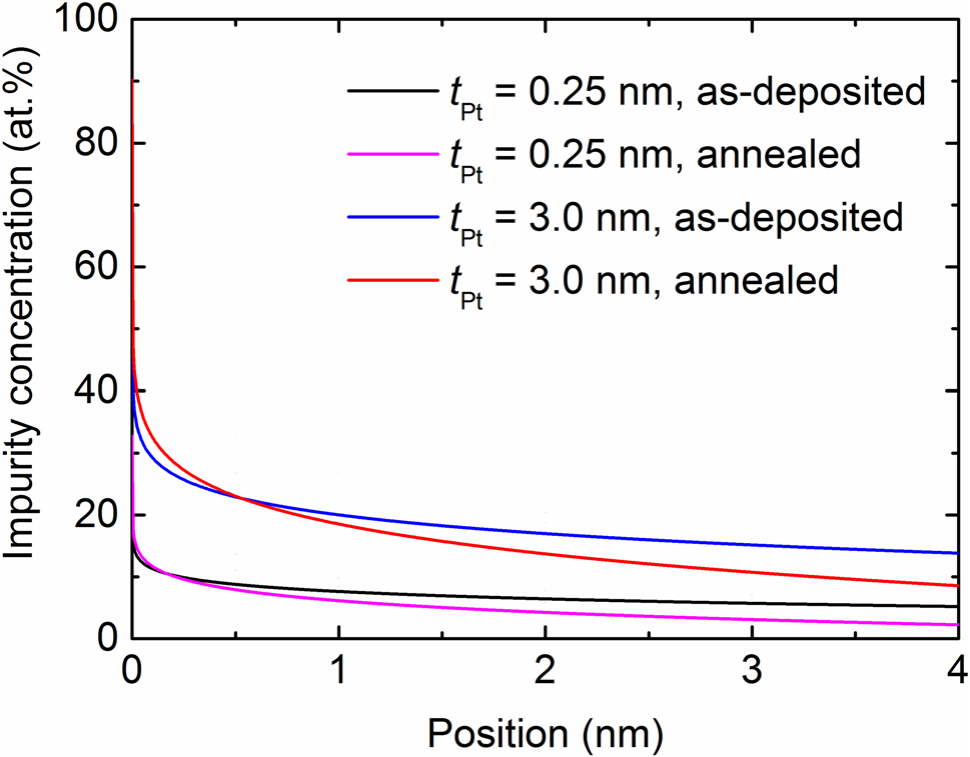
Results for the impurity (Pt or Ru or both) contents as a function of the position in the Co region at Pt thicknesses of 0.2 nm and 3.0 nm in the as-deposited and annealed samples.

Here, *M*_s,Co_ and *M*_s,sample_ are, respectively, *M*_s_ values of a pure Co and the Co layer interpenetrated with impurity atoms. *A*_imputiry_ indicates the decrease of *M*_s_ with an addition of 1 atomic percent impurity, whereas *C*_impurity_ denotes the impurity composition. Figure 4 shows the calculated results for the concentration of interpenetrated impurity atoms in the Co layer. The results are related to the position in the Co/Pt interface. As observed in Fig. 4, the concentration of interpenetrated impurities in the Co layer is not small. At the interface, the impurity level for the samples with *t*_Pt_ = 3.0 nm is as high as 37.2 at.% (as-deposited) or 43.7 at.% (annealed). The impurity level is lower for the sample with *t*_Pt_ = 0.25 nm, which is 14.5 at.% (as-deposited) or 16.2 at.% (annealed). The impurity concentrations of the annealed samples with *t*_Pt_ = 0.25 and 3.0 nm are higher than those of the as-deposited ones, indicating the diffusion of impurity atoms from the neighboring layers during annealing. Another possibility is the de-mixing of interpenetrated impurities from the deep region. The crossover occurs at positions of 0.2 and 0.5 nm for the samples with *t*_Pt_ = 0.25 and 3.0 nm, respectively. The crossover should depend on the amount of the interpenetrated atoms and the diffusion upon annealing. Although the crossover points are of interest, we do not performed a detailed analysis in this study, as our main concern is to know the level of impurity and its change upon annealing. As expected, the concentration of impurities decreases monotonically as it is located away from the interface. For example, at a position of 0.5 nm, which is relevant to the *t*_Co_ value in [Pt/Co] multilayers with the inverted structure^4,5^, the impurity concentration is 23 at.% for the samples with *t*_Pt_ = 3.0 nm. In contrast, at the same position, the impurity concentration is estimated to be 8 at.% for the samples with *t*_Pt_ = 0.25 nm, which is an optimum Pt thickness in the inverted [Pt/Co] multilayers. This explains the deteriorating effect of the interpenetrated Pt atoms on the PMA strength of the [Pt/Co] multilayers.

## Discussion

The effects of the top Pt layer thickness and annealing on the interface quality of [Pt/Co/Pt] trilayers were systematically investigated. Even with the cross-sectional HRTEM, it is difficult to identify the exact location of a very thin layer such as *t*_Pt_ = 0.25 nm. However, the HRTEM images, as shown in Fig. 1a–d, clearly show that the layer forms a continuous structure and its interfaces are atomically flat. These features are duly reflected by the magnetic properties and in this sense, the magnetometry can be a good tool to examine the interface properties of ultrathin magnetic films. From the MDL plots, the Co layer can be broken down into two parts that show different magnetic properties. In the high *t*_Co_ range, a linear behavior was observed. However, a nonlinear behavior was observed in the low *t*_Co_ range. Further, the proximity effect was detected in the low *t*_Co_ range. The nonlinearity in the *M* values can be converted to the Co-layer impurity concentration by using an analytical equation. The interpenetration and inter-diffusion depth were found to be sensitive to *t*_Pt_ and the annealing process. Specifically, for the samples with *t*_Pt_ = 0.25 nm, the impurity concentrations near the top Co/Pt interface are significantly smaller than that for the samples with *t*_Pt_ = 3.0 nm. This explains the relationship between the interface quality and PMA strength of the [Pt/Co] multilayers system. Although the impurity levels can vary depending on the assumptions made earlier, the relative impurity level is minimally affected by these assumptions. Therefore, the magnetometric investigation of the interfacial properties will aid in analyzing the interface quality of the magnetic multilayers system.

## Methods

The stack structure examined in this study consisted of the following: Si substrate (wet-oxidized)/Ta (5 nm)/Pt (10 nm)/Ru (30 nm)/Pt (3 nm)/Co (*t*_Co_)/Pt (*t*_Pt_)/Ru (3 nm). The two variables were *t*_Co_ (the thickness of the Co layer between the two Pt layers) and *t*_Pt_ (the thickness of the Pt layer on top of the Co layer). Thickness *t*_Co_ varied between 0.5 and 10 nm, whereas *t*_Pt_ was fixed at 0.25 or 3 nm. The samples were fabricated using an ultrahigh vacuum magnetron sputtering system with a base pressure of 8 × 10^−8^ Torr. All the layers were deposited at a constant Ar pressure of 2 × 10^−3^ Torr. No specific substrate cooling or heating was applied during the sputtering process. The thicknesses of the constituent layers were measured using a surface profiler. The deposition rate of the layers was adjusted to ~ 0.03 nm/s by varying the sputtering power. This deposition rate was used to calculate the thicknesses of the layers. The samples were annealed at 400 °C for 1 h under a vacuum pressure of 1 × 10^−6^ Torr. The magnetic moment was measured using a vibrating sample magnetometer (VSM), and the microstructure was examined using high-resolution transmission electron microscopy (HRTEM).
